# Elementary screening of lymph node metastatic-related genes in gastric cancer based on the co-expression network of messenger RNA, microRNA and long non-coding RNA

**DOI:** 10.1590/1414-431X20176685

**Published:** 2018-02-26

**Authors:** Zhonghua Song, Wenhua Zhao, Danfeng Cao, Jinqing Zhang, Shouhua Chen

**Affiliations:** 1 Department of Oncology, Shandong Jiaotong Hospital, Jinan, Shandong Province, China; 2 Department of Oncology, Shandong Provincial Qianfoshan Hospital, Shandong University, Jinan, Shandong Province, China; 3 Department of Obstetrics, Shandong Provincial Qianfoshan Hospital, Shandong University, Jinan, Shandong Province, China; 4 Department of Breast and Thyroid Surgery, Shandong Jiaotong Hospital, Jinan, Shandong Province, China; 5 Department of General Surgery, Shandong Provincial Qianfoshan Hospital, Shandong University, Jinan, Shandong Province, China

**Keywords:** Gastric cancer, Lymph node metastasis, Co-expression network, Modular analysis

## Abstract

Gastric cancer (GC) is the fifth most common cancer and the third leading cause of cancer-related deaths worldwide. The high mortality might be attributed to delay in detection and is closely related to lymph node metastasis. Therefore, it is of great importance to explore the mechanism of lymph node metastasis and find strategies to block GC metastasis. Messenger RNA (mRNA), microRNA (miRNA) and long non-coding RNA (lncRNA) expression data and clinical data were downloaded from The Cancer Genome Atlas (TCGA) database. A total of 908 differentially expressed factors with variance >0.5 including 542 genes, 42 miRNA, and 324 lncRNA were screened using significant analysis microarray algorithm, and interaction networks were constructed using these differentially expressed factors. Furthermore, we conducted functional modules analysis in the network, and found that yellow and turquoise modules could separate samples efficiently. The groups classified in the yellow and turquoise modules had a significant difference in survival time, which was verified in another independent GC mRNA dataset (GSE62254). The results suggested that differentially expressed factors in the yellow and turquoise modules may participate in lymph node metastasis of GC and could be applied as potential biomarkers or therapeutic targets for GC.

## Introduction

Gastric cancer (GC) is the fifth most common cancer and the third leading cause of cancer-related deaths worldwide (1). The high mortality of GC might be attributed to delay in detection and is closely related to metastasis and recurrence. Lymph node metastasis occurs in 70% of patients with advanced GC (2,3). It is an early event in GC metastasis and an independent prognostic factor, and can significantly affect the prognosis of patients (4). Therefore, predicting, diagnosing and investigating lymph node metastasis in GC is very important for the prognosis and treatment of patients.

The molecular mechanism of lymph node metastasis has been preliminarily clarified, and mainly includes cell migration and degradation of extracellular matrix, tumor cell apoptosis and immune escape, formation of new lymphatic vessels, and other aspects (5,6). A series of growth factors, cytokines, chemokines, miRNA and long non-coding RNA (lncRNA) associated with lymph node metastasis have been discovered, which were found to interact with each other to form a complex regulatory network and are involved in various processes of lymph node metastasis in GC (7–13). For example, miRNA-375 is downregulated in gastric carcinomas and regulates cell survival by targeting PDK1 and 14-3-3ζ (14), miRNA-7 functions as an anti-metastatic miRNA in GC by targeting insulin-like growth factor-1 receptor (15), and miR-148a contributes to the maintenance of homeostasis in normal stomach tissue and plays an important role in GC invasion by regulating MMP7 expression. (16). As for lncRNAs, the HOTAIR functions as a competing endogenous RNA to regulate HER2 expression by sponging MiR-331-3p (12), HMlincRNA717 may play crucial roles during cancer occurrence and progression (8 [Bibr B09]), and ATB plays an important role in epithelial-mesenchymal transition to promote invasion and metastasis through the TGFβ/miR-200s/ZEB axis, resulting in a poor prognosis in GC (10 [Bibr B11]). Although researchers have explored the factors that affect lymph node metastasis, most of the studies focus on one or several factors, and the molecular mechanism of lymph node metastasis is still unclear.

Recent developments in bioinformatics and statistical genomics provide biological systems approaches to better understand the organization of the transcriptome and transcriptional regulation. Among all of the systematic biology approaches, gene network analysis is a powerful approach that considers gene interactions. It has been widely applied in gene expression studies of humans and model organisms (17–19).

In the present study, we used large quantities of messenger RNA (mRNA)-seq and microRNA (miRNA)-seq data in GC patients in The Cancer Genome Atlas (TCGA) database to screen mRNA, miRNA, and lncRNA with differential expression between the samples with and without lymph node metastasis, and then construct the co-expression networks based on the differential expression of various factors. The network module and Cox regression model were combined to screen survival-related genes. Moreover, the correlation between different expression levels of these genes and prognosis of GC patients was verified in another independent dataset.

## Material and Methods

### Data sources

Samples of gastric cancer were selected from the TCGA database (http://cancergenome.nih.gov/), in which mRNAs data and miRNAs data were profiled from the Illumina platform. The lncRNAs data were annotated from mRNA transcriptomic database through matching to the HGNC database. In total, 396 samples with mRNA-miRNA-lncRNA paired samples were obtained, and 356 samples remained after removing 40 samples with unclear status in lymph node metastasis. A total of 210 lymph node metastasis samples and 146 non-lymph node metastasis samples were included.

### Data preprocessing

After download from TCGA, the expression profiles in level 3 were merged and prepared. The mRNA, miRNAs and lncRNAs with ≥20% missing values were removed, while those with <20% missing values were replaced by mean values. The values of lncRNA and mRNA were assessed by PRKM, and miRNA values were assessed by RPM. Next, these values were transformed by log2 logarithms to obtain Gaussian distribution.

### Identification of differentially expressed genes and functional enrichment analysis

Significant analysis microarray (SAM) (20) is an algorithm used to screen the differentially expressed genes (DEGs). When differential gene expression was simply checked by *t*-test or variance analysis, high rates of false positive results were produced under repeated tests. However, SAM is effective at correcting the false positive rates through controlling the false discovery rate (FDR) in multiple tests to filter DEGs with significant differences. An absolute value of log2 ratio ≥1.5 and FDR ≤0.001 were set as the threshold for determining the significance of gene expression difference.

The identified DEGs were analyzed in terms of gene ontology (GO) function and pathway enrichment analysis using the DAVID (Database for Annotation, Visualization, and Integrated Discovery) hypergeometric test (21).

### Construction of co-expression networks and excavation of network modules

Based on information of disease-related gene expression profiles under GC, co-expression networks were constructed by calculating adjacency matrix A of a gene pair using the WGCNA package (<https://cran.r-project.org/web/packages/WGCNA/index.html>) (22). To calculate the adjacency matrix, an intermediate quantity called the co-expression similarity s_ij_ was first defined. The default method defines the co-expression similarity s_ij_ as the absolute value of the correlation coefficient between the profiles of nodes i and j:

(Equation 1) $${\text{S}}_{\text{ij}}=|\text{cor}\left({\text{X}}_{\text{i}},{\text{X}}_{\text{j}}\right)|$$

where x_i_ and x_j_ are the vector expression values of gene i and j, respectively, and Cor is used to evaluate Pearson correlation coefficient of the two vector values.

A weighted network adjacency was defined by raising the co-expression similarity to a power:

(Equation 2) $${\text{a}}_{\text{ij}}=S_{\text{ij}}^{\beta }$$

With β≥1, the adjacency function calculates the adjacency matrix from expression data. The adjacency in Equation 2 implies that the weighted adjacency a_ij_ between two genes is proportional to their similarity on a logarithmic scale, log(a_ij_) = β × log(s_ij_). Pearson correlation coefficient S_ij_ is exponentially transformed into connection coefficient a_ij_, to achieve a reliable network.

Network topology property is taken into account to excavate modules of co-expression networks in WGCNA. This algorithm analyses not only the relationship of two conjoint node genes, but other genes correlated to the two nodes. Connection coefficient a_ij_ in co-expression network was turned into weight coefficient W_ij_ by the following formula:

(Equation 3) $${\text{W}}_{\text{ij}}=\frac{{\text{I}}_{\text{ij}}+{\text{a}}_{\text{ij}}}{\min\left\{{\text{k}}_{\text{i}}{\text{k}}_{\text{j}}\right\}+1-{\text{a}}_{\text{ij}}}$$

$${\text{of which, I}}_{\text{ij}}=\sum {}_{\text{u}}{\text{a}}_{\text{iu}}{\text{a}}_{\text{uj}}{\text{k}}_{\text{i}}=\sum {}_{\text{u}}{\text{a}}_{\text{iu}}$$

W_ij_ considers overlapping between neighbors of the two conjoint genes i and j. Network modules were excavated after hierarchical cluster analysis of W gene weighted matrix.

### Survival analysis

Gastric carcinoma related genes were sorted out on the background of expression profile data, and then were constructed into the co-expression network according to their expression levels, which was next divided into various modules. Samples were hierarchically clustered based on module genes and the significant differences of survival time between samples were analyzed through K-M simple clustering. Finally, analysis of COX single variable regression (23) was carried out between survival time and factors in modules.

## Results

### Preprocessing of expression profile data

**Figure 1.**
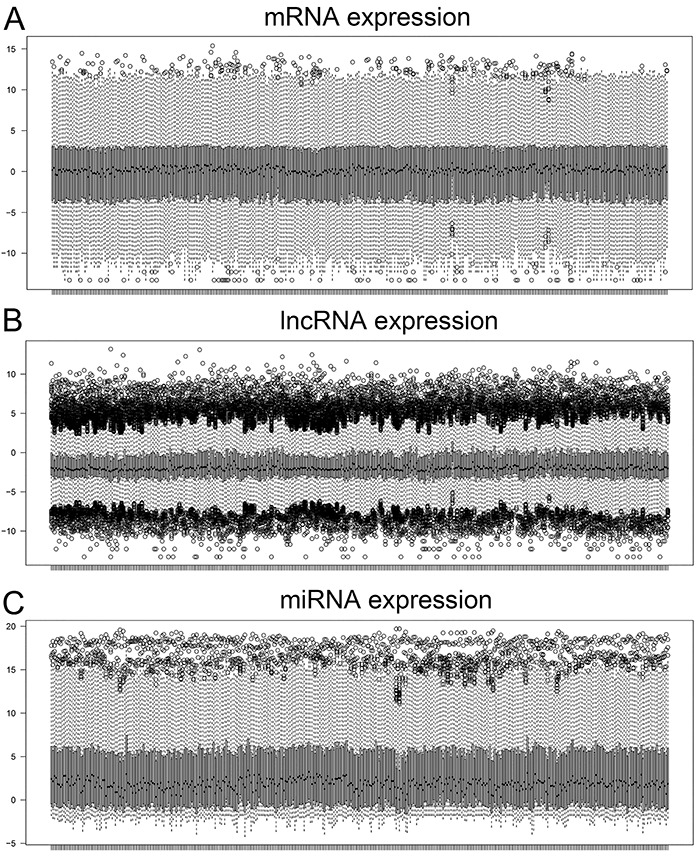
Box plot distribution of TCGA data related to gastric carcinoma samples. *A*, messenger RNA (mRNA) expression; *B*, long non-coding RNA (lncRNA) expression; *C*, microRNA (miRNA) expression. Horizontal axis indicates cancer samples; vertical axis indicates the expression value distribution of the mRNAs, lncRNAs and miRNAs.

There were 560 miRNAs and 12,474 mRNAs left after preprocessing. Totally 1,165 lncRNAs were obtained from transcripts of mRNA expression profiles matched to HGNC database (http://www.genenames.org/). The expression data of mRNA, lncRNA and miRNA after preprocessing are displayed in Figure 1. After standardization, the distribution values among samples were relatively uniform.

### Screening of differentially expressed factors and functional enrichment analysis

**Figure 2.**
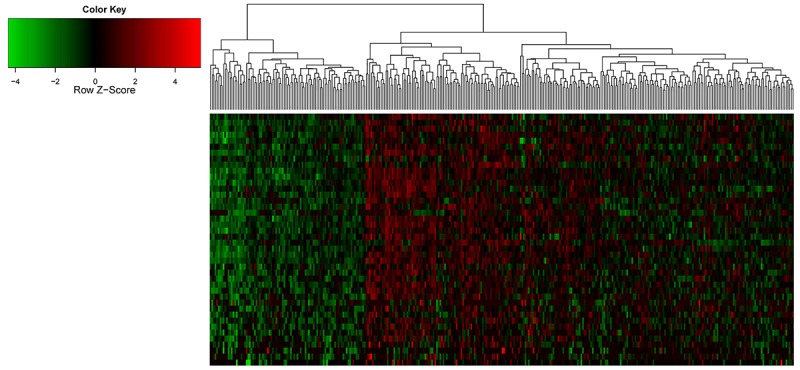
Heatmap of sample clustering based on differentially expressed factors. Horizontal axis indicates samples; vertical axis indicates differentially expressed factors.

The differentially expressed mRNA, miRNA and lncRNA were screened by SAM algorithm. The result showed that 908 differentially expressed factors with variance >0.5 were screened, including 324 differentially expressed lncRNAs, 42 differentially expressed miRNAs and 542 differential expressed genes (see Supplementary Table S1). These differentially expressed factors divided the samples into two classes as shown in Figure 2.

In all, 542 DEGs were functionally enriched by DAVID analysis. There were 15 major enriched GO terms assigned into the biological process categories, including immune response (GO:0006955), response to stress (GO:0006950), defense response (GO:0006952), cell surface receptor signaling pathway (GO:0007166), cell proliferation (GO:0008283), regulation of immune system process (GO:0002682), cell migration (GO:0016477), leukocyte activation (GO:0045321), inflammatory response (GO:0006954), positive regulation of response to stimulus (GO:0048584), regulation of cell proliferation (GO:0042127), cell differentiation (GO:0030154), cellular developmental process (GO:0048869), cell adhesion (GO:0007155), and biological adhesion (GO:0022610) (Table 1, also see Supplementary Table S2).

**Table 1.** Top 15 gene ontology functions enriched by differently expressed genes.

ID	Description	P value	P adjusted
GO:0006955	Immune response	2.19E-63	3.27E-61
GO:0006952	Defense response	2.15E-57	2.80E-55
GO:0006950	Response to stress	1.57E-56	1.96E-54
GO:0007166	Cell surface receptor signaling pathway	1.20E-55	1.43E-53
GO:0008283	Cell proliferation	1.06E-49	1.09E-47
GO:0002682	Regulation of immune system process	6.12E-42	4.82E-40
GO:0016477	Cell migration	7.58E-40	5.66E-38
GO:0045321	Leukocyte activation	1.90E-39	1.32E-37
GO:0006954	Inflammatory response	3.92E-38	2.66E-36
GO:0048584	Positive regulation of response to stimulus	6.10E-38	4.05E-36
GO:0042127	Regulation of cell proliferation	1.72E-37	1.10E-35
GO:0030154	Cell differentiation	3.01E-34	1.70E-32
GO:0048869	Cellular developmental process	2.06E-33	1.14E-31
GO:0007155	Cell adhesion	7.77E-33	4.22E-31
GO:0022610	Biological adhesion	1.11E-32	5.90E-31

Fisher's exact test was used for statistical analyses, and the P value was adjusted by Bonferroni correction.

### Construction of co-expression networks and mining of modules

**Figure 3.**
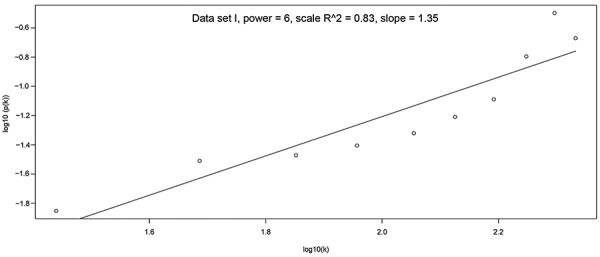
Gene degrees in co-expression network and distribution of corresponding numbers. Horizontal axis indicates values of gene degrees (k); vertical axis indicates proportion of gene with k degree, that is, p (k).

Based on the above mentioned 908 differential factors (including differentially expressed genes, miRNA and lncRNA), co-expression networks were constructed by using WGCNA package in R language. The gene degrees in the co-expression network were submitted to the power distribution law (24), which showed in line with the free scale characteristic in biological networks (Figure 3).

**Figure 4.**
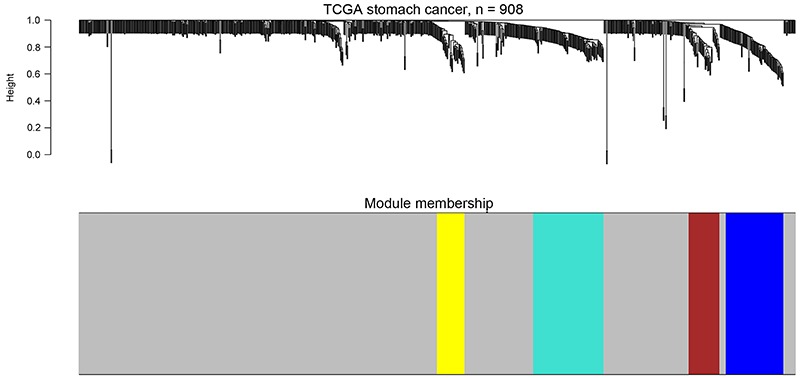
Clustering results of gene modules in co-expression network. *Top*, hierarchical clustering of 908 genes in co-expression network. *Bottom*, divisional module classes. Gray zones indicate genes that do not belong to any modules.

Furthermore, we carried out the module mining of genes in the co-expression networks. As shown in Figure 4, these differentially expressed factors in the co-expression networks were divided into four module groups presented by blue, brown, turquoise and yellow color, which included 73, 39, 89, and 35 differentially expressed factors, respectively. In addition, there were other 672 factors that could not be modular, and were presented in grey.

### Classification validation of factors in four modules

**Figure 5.**
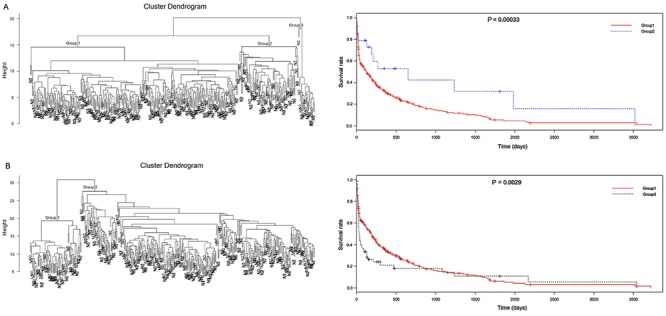
Sample clustering based on yellow (*A*) and turquoise (*B*) module factors and differences in survival curves of the two groups of samples.

The following analysis was the classification of 356 samples based on the module factors. It was found that yellow and turquoise modules could separate samples efficiently (group 3 was excluded when classified by yellow module because there were rare death samples in the group), and there were significant differences between the survival curves of isolated samples (P<0.01; Figure 5). By contrast, the genes in blue and brown modules could not separate samples effectively, and therefore, these genes were given up in the following survival time analysis.

Further validation between factors in yellow and turquoise modules and survival time of GC samples was performed by COX univariate regression analysis. The top 7 factors with significant regression in yellow module are shown in Table 2, including adenosine receptor A3 (ADORA3), toll-like receptor 7 (TLR7), interferon regulatory factor (IRF4), CC chemokine receptor 4 (CCR4), reticulon-1 (RTN1), growth factor receptor-bound protein 2 (GRB2)-binding adaptor protein (GAPT), and GRB2-related adapter protein 2 (GRAP2). There were 10 factors with significant regression in turquoise module, including six genes (guanine nucleotide-binding protein G(o) subunit alpha, GNAO1; isthmin-1, ISM1; cartilage intermediate layer protein, CILP; slit homolog 2 protein, SLIT2; scrapie-responsive protein 1, SCRG1; tumor necrosis factor α-induced protein 8 (TNFAIP8)-like protein 3, TNFAIP8L3), two miRNAs (hsa-mir-183 and hsa-mir-942) and two lncRNA (MIR345 and HCG18) (Table 2).

**Table 2.** Genes with top significance of COX univariate regression in the yellow and turquoise modules.

Gene symbol	P value	Module
ADORA3	1.64E-05	Yellow
TLR7	1.84E-03	Yellow
IRF4	1.86E-03	Yellow
RTN1	3.68E-03	Yellow
CCR4	3.42E-02	Yellow
GAPT	3.78E-02	Yellow
GRAP2	4.05E-02	Yellow
GNAO1	9.94E-07	Turquoise
hsa-mir-942	1.17E-05	Turquoise
ISM1	3.55E-05	Turquoise
CILP	3.98E-05	Turquoise
SLIT2	7.82E-05	Turquoise
MIR345	9.46E-05	Turquoise
hsa-mir-183	1.88E-04	Turquoise
SCRG1	2.67E-04	Turquoise
TNFAIP8L3	4.84E-04	Turquoise
HCG18	5.58E-04	Turquoise

### Classification validation of factors in yellow and turquoise modules

**Figure 6.**
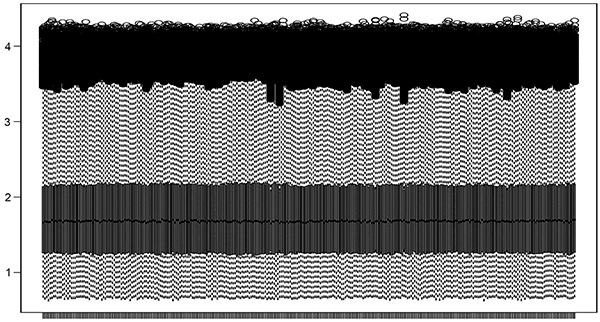
Box plot distribution of GSE62254 dataset.

GSE62254, the expression profile dataset of gastric cancer, was obtained from GEO database (http://www.ncbi.nlm.nih.gov/geo/query/acc.cgi?acc=GSE62254), including survival time information of 300 samples, which generated from the GPL570 platform. In the pretreated samples, 20,692 genes were found and their box plot distribution is shown in Figure 6.

**Figure 7.**
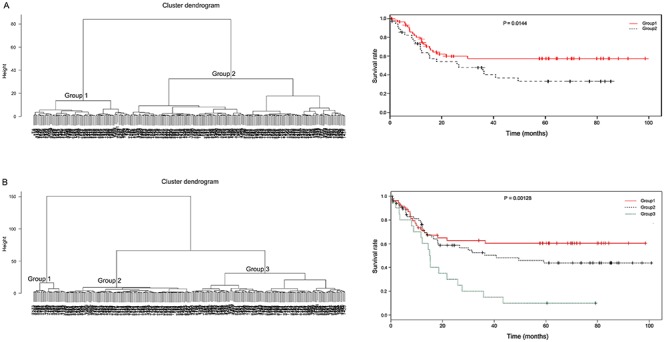
Clustering of gastric cancer samples in GSE62254 dataset based on genes in yellow (*A*) and turquoise (*B*) modules.

Thirty-four genes within yellow module were gained from the GSE62254 database. Using these module genes, samples were well grouped into two classes. Moreover, the survival time in Figure 7A showed significant differences between the two groups of samples (P=0.0144). The similar results were received via 81 genes of turquoise module contained in GSE62254 database: samples were divided into three groups, and their survival times exhibited significant differences (P=0.00128; Figure 7B). These analyses demonstrated that selected genes of yellow and turquoise modules have significant correlation with survival time of GC samples.

## Discussion

Lymph node metastasis and recurrence are the main factors that affect the prognosis of GC, and lymph nodes without metastasis have important immune monitoring functions. Therefore, it is very important to accurately determine the extent and degree of lymph node metastasis, and to carry out rational lymph node dissection. As an early and complicated event in GC metastasis, lymph node metastasis involves a series of functional and regulating genes (4), and therefore it is more meaningful to mine the co-expression network of various cancer related factors. In the present study, we downloaded large quantities of mRNA-seq and miRNA-seq data from the TCGA database to screen the mRNA, miRNA and lncRNA related to GC lymph node metastasis, and then constructed co-expression networks based on the differential expression of these factors. Compared with previous studies, which only focused on one or two factors of coding genes, miRNAs and lncRNAs (7–10,15,25–27), the present study performed a systematic analysis of the three factors for the first time. There were 542 DEGs which were functionally enriched into 15 major GO terms in the biological process category, most of which were related to cancer, such as immune response, cell proliferation, cell migration, cell differentiation and cell adhesion.

Thanks to the rapid development in bioinformatics and statistical genomics, gene network analysis has become a powerful approach that can explore the interactions between genes and has been widely applied in gene expression studies of humans and model organisms (17–19). Meanwhile, genes that are highly interconnected within the network are usually involved in the same biological modules or pathways, and therefore, modular analysis also plays an important role in the analysis of gene co-expression network (28,29). Several studies have demonstrated the value of analyzing networks based on TCGA database. In the present study, 908 differentially expressed factors including 542 genes, 42 miRNAs and 324 lncRNAs were included in the network analysis, and furthermore, genes in the co-expression networks were used for the modular mining. Four module groups were coded in blue, brown, turquoise and yellow color, which included 73, 39, 89, and 35 differentially expressed factors. Except genes in the group 3 of the yellow module lacking sufficient death number, the other genes in yellow and turquoise modules could separate samples efficiently, and there were significant differences between the survival curves of isolated samples.

Based on the network and modular analysis, seven (ADORA3, TLR7, IRF4, CCR4, RTN1, GAPT, and GRAP2) and ten (GNAO1, ISM1, CILP, SLIT2, SCRG1, TNFAIP8L3, hsa-mir-183, sa-mir-942, MIR345 and HCG18) candidate factors with top significance of COX univariate regression were identified in the yellow and turquoise module, respectively. Within yellow module, ADORA3 (30), TLR7 (31), IR4 (32), and CCR4 (33–35) are genes closely related to lymph node metastasis and survival of GC, and within turquoise module, two miRNAs (miRNA-183 and miRNA-942) (13,36) and three genes (GNAO1, ISM1and SLIT2) are factors involved in the regulation of GC. The above published studies prove that our gene network analysis for screening candidate factors related to GC for further evaluation was reliable.

Therefore, the remaining seven identified factors including three genes in yellow module (RTN1, GAPT, GRAP2) and five factors in turquoise module (CILP, SCRG1, TNFAIP8L3, MIR345 and HCG18) could be new factors related to survival of GC. In fact, there is evidence that these genes may be associated with several diseases and even cancer. For example, RTN1, a neuroendocrine cell specific protein, localized in endoplasmic reticulum, might be involved in the activation of the expression of androgen-responsive genes and related to prostate cancer (37). As an adapter protein, GRB2 has been identified as a major mediator in Ras-mitogen-activated protein kinase (MAPK) activation, which is essential for growth factor-induced cell proliferation and differentiation and plays a central role in embryo development and malignant transformation. Therefore, we believe that GAPT and GRAP2 are involved in the activation of MAPK and growth factor-induced cell proliferation and differentiation, which are often associated with the development of cancer (38). CILP, an extracellular matrix protein abundant in cartilaginous tissues, is implicated in common musculoskeletal disorders, including osteoarthritis and lumbar disc disease (39). It is worth noting that the TNFAIP8 family members are usually associated with immune homeostasis and inflammatory cancer diseases. For example, TNFAIP8 itself usually functions as an oncogenic molecule and it is also associated with enhanced cell survival and inhibition of apoptosis, and TNFAIP8-like 2 (TIPE2) governs immune homeostasis in both the innate and adaptive immune system and prevents hyper-responsiveness (40). However, the function of TNFAIP8L3 remains unclear. Therefore, our study provides some insight into the emergent properties of prognostic genes, and further investigation of the functional roles of these newly identified factors is urgent for a functional validation system in GC.

In conclusion, our data provides a comprehensive bioinformatics analysis of genes and pathways, which may be involved in the lymph node metastasis of GC. We found a total of 542 genes, 42 miRNAs and 324 lncRNAs, and then constructed the interaction networks of these differentially expressed factors. Furthermore, we conducted functional modules analysis in the network, and found that except for the genes in group 3 of the yellow module, the other genes in the yellow and turquoise modules could separate samples, and therefore these genes could be potential prognostic biomarkers. However, further analyses are still required to reveal their mechanism in the process of tumor genesis and development in GC.

## Supplementary Material

Click here to view [pdf].
